# From Echocardiography to CT/MRI: Lessons for AI Implementation in Cardiovascular Imaging in LMICs—A Systematic Review and Narrative Synthesis

**DOI:** 10.3390/bioengineering12101038

**Published:** 2025-09-27

**Authors:** Ahmed Marey, Saba Mehrtabar, Ahmed Afify, Basudha Pal, Arcadia Trvalik, Sola Adeleke, Muhammad Umair

**Affiliations:** 1 Department of Radiology, Sheikh Khalifa Medical City, Abu Dhabi 51900, United Arab Emirates; ahmed.ahmed1797@alexmed.edu.eg; 2 Department of Radiology, Tabriz University of Medical Sciences, Tabriz 5178654714, Iran; 3 Faculty of Medicine, Benha University, Benha 13518, Egypt; 4 Department of Electrical and Computer Engineering, The Johns Hopkins University, Baltimore, MD 21218, USA; 5 Department of Emergency Medicine, Washington Hospital Center, MedStar Health, Washington, DC 20010, USA; 6 School of Biomedical Engineering and Imaging Sciences, King’s College London, London SE1 7EH, UK; s.adeleke@alumni.ucl.ac.uk; 7 Russell H. Morgan Department of Radiology and Radiological Sciences, The Johns Hopkins Hospital, Baltimore, MD 21287, USA; mu2331@cumc.columbia.edu; 8 Department of Radiology, Columbia University Irving Medical Center, New York, NY 10032, USA

**Keywords:** artificial intelligence, cardiovascular imaging, low- and middle-income countries (LMICs), healthcare disparities, digital health, resource-limited settings

## Abstract

**Objectives**: The aim of this study was to synthesize current evidence on artificial intelligence (AI) adoption in cardiovascular imaging across low- and middle-income countries (LMICs), highlighting diagnostic performance, implementation barriers, and potential solutions. **Methods**: We conducted a systematic review of PubMed, Embase, Cochrane Library, Web of Science, and Scopus for studies evaluating AI-based echocardiography, cardiac CT, or cardiac MRI in LMICs. Articles were screened according to PRISMA guidelines, and data on diagnostic outcomes, challenges, and enabling factors were extracted and narratively synthesized. **Results**: Twelve studies met the inclusion criteria. AI-driven methods frequently surpassed 90% accuracy in detecting coronary artery disease, rheumatic heart disease, and left ventricular hypertrophy, often enabling task shifting to non-expert operators. Challenges included limited dataset diversity, operator dependence, infrastructure constraints, and ethical considerations. Insights from high-income countries, such as automated segmentation and accelerated imaging, suggest potential for broader AI integration in cardiac MRI and CT. **Conclusions**: AI holds promise for enhancing cardiovascular care in LMICs by improving diagnostic accuracy and workforce efficiency. However, multi-center data sharing, targeted training, reliable infrastructure, and robust governance are essential for sustainable adoption. This review underscores AI’s capacity to bridge resource gaps in LMICs, offering practical pathways for future research, clinical practice, and policy development in global cardiovascular imaging.

## 1. Introduction

Cardiovascular diseases (CVDs) are the leading cause of global mortality, with low- and middle-income countries (LMICs) bearing over 80% of CVD deaths [1,2]. Advances in echocardiography, functional testing, CT, and MRI have improved outcomes in high-income settings, but many LMICs face critical barriers: limited imaging infrastructure, radiologist shortages, and inadequate funding. For instance, Yuyun et al. highlight the disproportionate CVD burden in Sub-Saharan Africa compared with high-income countries, emphasizing the structural inequities in healthcare delivery [3]. Yan et al. discuss the emerging, but still underutilized, role of AI in cardiovascular medicine, pointing to its potential for filling diagnostic gaps [4]. Meanwhile, Moosavi et al. provide one of the first large-scale prospective validations of AI interventions in cardiology, confirming that such tools can achieve clinically reliable performance [5]. Complementing these perspectives, Qin et al. demonstrate the feasibility of sustainable low-field cardiovascular MRI as an affordable alternative for changing healthcare systems in LMICs [6].

The disparity in resources is pronounced. Sub-Saharan Africa possesses approximately 0.3 MRI units per million population, in stark contrast to nearly 30 units per million in high-income countries [7]. The shortage of trained personnel is equally critical: Tanzania, with a population of 58 million, has only about 60 radiologists [8]. As emphasized by Mollura et al., this represents nearly fifty times fewer radiologists per capita than in the United States, with comparable deficiencies evident across other subspecialties, including breast imaging in Kenya [9]. Such inequities highlight the urgent necessity for scalable artificial intelligence solutions capable of improving access, diagnostic accuracy, and the overall utility of cardiovascular imaging in resource-constrained environments.

AI has already shown promise across multiple cardiovascular applications. For example, Hanneman et al. emphasize its ability to create value in CVD diagnosis and risk stratification [10], while Lim et al. discuss its integration into cardiovascular imaging workflows for efficiency and precision [11]. Beyond cardiology, Hosny et al. illustrate how AI in radiology can improve diagnostic accuracy by reducing human variability [12], and Khalifa et al. highlight its role in clinical prediction and personalized treatment planning [13]. Together, these studies show that AI can improve diagnostic accuracy in coronary artery disease, heart failure, and rheumatic heart disease while reducing reliance on invasive or costly procedures.

However, deploying AI in LMICs encounters substantial obstacles. Alami et al. frame the infrastructural, regulatory, and social prerequisites for responsible AI adoption in global health [14]. The Lancet Public Health editorial underscores the need for fairness and precision in public health applications of AI [15]. Khan et al. specifically address the contextual challenges faced by low-income countries, from workforce shortages to lack of digital ecosystems [16]. On the clinical side, Ahmed et al. document the limited knowledge and readiness of medical students and doctors in Pakistan to use AI in practice [17]. Fletcher et al. raise concerns about fairness and bias in global health AI [18], while Hanna et al. explore the ethical and medicolegal implications of algorithm deployment [19]. Collectively, these works emphasize that insufficient infrastructure, workforce shortages, cultural barriers, and reliance on high-income datasets continue to limit AI’s generalizability in LMIC populations.

Despite these challenges, evidence of AI’s potential in LMIC cardiovascular imaging is growing. Brown et al. show that portable AI-driven echocardiography can significantly improve rheumatic heart disease detection in school-based screening programs [20]. Providência et al. affirm that handheld echocardiography, augmented by AI, offers a practical and scalable tool for rheumatic heart disease screening [21]. Similarly, Farina et al. demonstrate that AI applied to chest radiography enables cost-effective CVD identification in underserved populations [22]. As Mollura et al. argue, collaborative training programs can further empower a limited radiology workforce to manage high imaging volumes [9]. These advances highlight how AI, when paired with sustainable infrastructure and locally tailored algorithms, can address healthcare inequities. In related medical domains, algorithms have also been used to identify problematic or non-generalizable training images, such as in dermatology, in which AI systems flag inputs that would likely fail during inference [23]. Extending this approach to cardiovascular imaging could help ensure that models trained on high-income datasets maintain robust performance when applied to LMIC populations.

**Figure 1:**
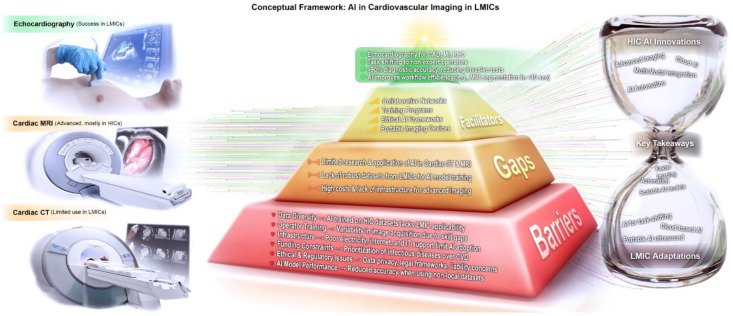
Conceptual framework for AI use in cardiovascular imaging in LMICs.

While awareness of AI’s capabilities is growing, comprehensive evaluations in LMIC contexts remain scarce. This systematic review therefore examines (1) the current landscape of AI-driven cardiovascular imaging in LMICs, (2) key barriers to adoption and implementation, and (3) strategic solutions for equitable, scalable deployment. Figure 1 delineates our conceptual framework, which synthesizes the interrelated barriers, gaps, and facilitators of AI-driven cardiovascular imaging, and elucidates how innovations originating in high-income countries may be strategically reconfigured to address the unique challenges of LMIC contexts.

## 2. Methods

### 2.1. Protocol Registration and Study Design

This systematic review was conducted in accordance with the Preferred Reporting Items for Systematic Reviews and Meta-Analyses (PRISMA) guidelines [24]. The review protocol was registered in PROSPERO (registration number: CRD42025634337).

### 2.2. Search Strategy

PubMed, Embase, Cochrane Library, Web of Science, and Scopus were searched through 5 January 2025, using MeSH terms and keywords for “cardiovascular diseases”, “cardiac imaging”, “artificial intelligence”, and “LMICs” (Supplementary Table S1). Only English-language publications were included.

### 2.3. Study Selection

Two reviewers independently screened titles and abstracts, removed duplicates, and resolved disagreements via a third reviewer. Full texts of potentially relevant articles were then assessed against the inclusion/exclusion criteria (Table 1).

### 2.4. Definition of AI

AI covers any computational technique employing machine learning (ML), deep learning (DL), or advanced algorithms to analyze data, identify patterns, or make predictions, including the following:

- Supervised ML (e.g., linear classifiers/regressors, decision trees, support vector machines);
- Unsupervised ML (e.g., clustering, dimensionality reduction);
- Hybrid/ensemble methods (e.g., bagging, boosting, stacking);
- Deep learning (e.g., convolutional neural networks, recurrent neural networks, transformers).

We excluded rule-based systems or static decision trees without learnable components and included only algorithms supporting iterative or data-driven learning, such as probabilistic or adaptive methods.

### 2.5. Data Extraction

Two authors independently extracted data via a standardized form on study characteristics (first author, year, country, population, sample size), imaging modality, AI model, objectives, main outcomes, and reported challenges; a third author resolved any discrepancies.

### 2.6. Data Synthesis

A narrative synthesis was performed due to heterogeneity in study designs, AI methods, and outcomes; no meta-analysis was conducted.

## 3. Results

### 3.1. Summary of Included Studies

Study characteristics (Table 2):

The twelve included studies spanned 2013–2024 and were geographically diverse, with representation from Iran, Pakistan, Ethiopia, South Africa, Vietnam, Uganda, Lesotho, Tunisia, and rural Australia. Most studies focused on echocardiography as the primary imaging modality, with additional work using phonocardiograms, Doppler echocardiography, and handheld point-of-care ultrasound (POCUS). Sample sizes ranged from fewer than 100 patients to large, community-based surveys exceeding 700 participants.

**Table 2:** Study characteristics.

First Author/Year	Population	Country	Imaging Modality
Alizadehsani (2013a) [25]	303 patients (Z-Alizadeh Sani dataset)	Iran	Echocardiography
Alizadehsani (2018) [26]	500 patients (extended Z-Alizadeh Sani dataset)	Iran	Echocardiography
Alizadehsani (2013b) [27]	303 patients (Z-Alizadeh Sani dataset)	Iran	Echocardiography + Lab features
Hoodbhoy (2018) [28]	525 pregnant women	Pakistan	Doppler echocardiography
Asmare (2021) [29]	124 RHD cases + 127 controls	Ethiopia	Phonocardiogram
Sayadi (2022) [30]	303 patients (Z-Alizadeh Sani dataset)	Iran	Echocardiography + Lab features
Ekambaram (2023) [31]	Patients with suspected valve emergencies	South Africa	Handheld POCUS (HoPE)
Nguyen (2023) [32]	109 public + 60 local patients	Vietnam	Echocardiography
Brown (2024) [20]	511 echocardiograms (282 RHD, 229 controls)	Uganda	Echocardiography + Doppler
Firima (2024) [33]	756 participants	Lesotho	Focused echocardiography
Soh (2024) [34]	157 participants	Australia (rural)	AI-guided TTE
Tromp (2024) [35]	94 patients	Tunisia	Nurse-led AI-POCUS

AI methods and objectives (Table 3):

The studies employed a broad spectrum of machine learning approaches. Support vector machines (SVMs) were the most frequently applied, particularly for CAD and RHD detection. Ensemble methods (bagging, boosting, decision trees, k-nearest neighbors) and deep learning architectures (CNNs, transformers) were increasingly adopted in more-recent work. Bayesian frameworks and logistic regression were used in specific clinical contexts such as valve emergencies and early CAD detection. The objectives largely centered on diagnostic prediction (CAD, MI, RHD, LVH) and proof-of-concept demonstrations for scalable screening tools in low-resource settings.

**Table 3:** AI methods and objectives.

First Author/Year	AI/ML Model	Objective
Alizadehsani (2013a) [25]	Bagging with SMO, neural networks, naïve Bayes	Non-invasive CAD diagnosis
Alizadehsani (2018) [26]	SVM, naïve Bayes, decision trees	Stenosis prediction (LAD, LCX, RCA)
Alizadehsani (2013b) [27]	Decision trees, bagging	Non-invasive coronary stenosis diagnosis
Hoodbhoy (2018) [28]	Multiple kernel learning	Predict adverse perinatal outcomes
Asmare (2021) [29]	SVM (RBF kernel)	Automated RHD screening via heart sounds
Sayadi (2022) [30]	Logistic regression, SVM	Early CAD detection with minimal features
Ekambaram (2023) [31]	Bayesian ML framework	Valve emergency diagnosis
Nguyen (2023) [32]	Ensemble learning (SVM, LR, DT, KNN)	MI detection
Brown (2024) [20]	SVM, CNNs, transformers	Automated RHD detection (MR analysis)
Firima (2024) [33]	Deep learning	LVH diagnosis with focused echo
Soh (2024) [34]	Deep learning	Valve disease and HF diagnosis in rural settings
Tromp (2024) [35]	Deep learning (AI-TRIO)	Nurse-led HF detection with AI-POCUS

Outcomes and challenges (Table 4):

Across conditions, the reported diagnostic accuracy was often high, with sensitivity and specificity frequently above 90%. For example, SVM-based CAD models achieved up to 96% accuracy, and phonocardiogram-based RHD screening reached 96% sensitivity and specificity. Nurse-led AI-guided POCUS demonstrated feasibility in detecting cardiac dysfunction with strong performance metrics. However, challenges were consistent across studies: small or single-center datasets, lack of external validation, dependence on operators for image acquisition, infrastructure constraints, and limited generalizability. Several studies also noted computational demands that could restrict real-world deployment in LMIC contexts.

**Table 4:** Outcomes and challenges.

First Author/Year	Outcome	Challenges
Alizadehsani (2013a) [25]	94.1% accuracy (SMO)	Limited dataset, reliance on manual features
Alizadehsani (2018) [26]	96.4% accuracy, 100% sensitivity, 88.1% specificity	Lack of clinical implementation, generalizability
Alizadehsani (2013b) [27]	79.5% LAD, 61.5% LCX, 69% RCA accuracy	Small dataset, no real-world testing
Hoodbhoy (2018) [28]	Protocol; results awaited	Synthetic data, need for external validation
Asmare (2021) [29]	96% sensitivity, 96% specificity, F1 = 96%	Imbalanced data, open-access quality concerns
Sayadi (2022) [30]	95.5% accuracy, 95.9% sensitivity, 91.7% specificity	Small dataset, no external validation
Ekambaram (2023) [31]	Improved time to diagnosis in valve emergencies	Handheld device limitations, operator dependency
Nguyen (2023) [32]	91.4% accuracy (public), 76.7% (local)	High computational needs, dataset localization issues
Brown (2024) [20]	AUC 0.93 (SVM), 0.84 (DL ensemble)	Limited aortic inclusion, device generalization issues
Firima (2024) [33]	87.5% evaluable images, 81.9% confirmed	Manual intervention required, quality maintenance
Soh (2024) [34]	42.3% with abnormalities detected	Variable image quality, novice dependency
Tromp (2024) [35]	92% sensitivity, 81% specificity	Small sample, nurse variability, limited generalization

**Figure 2:**
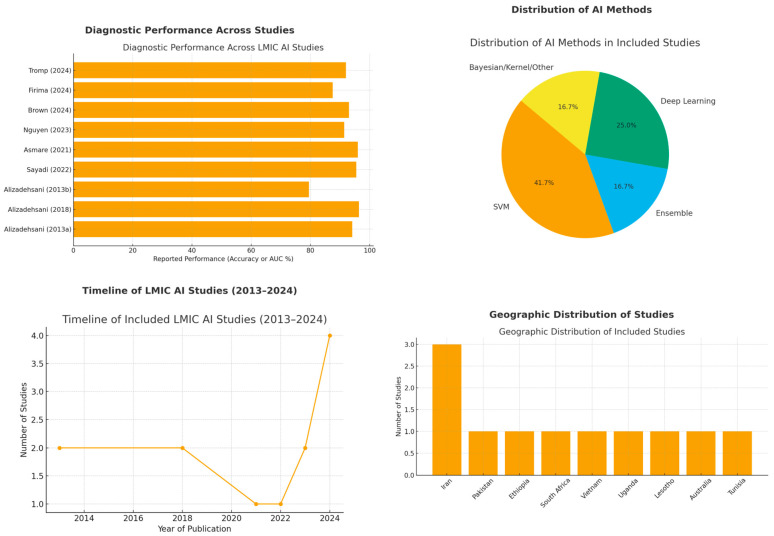
Visual analyses of included studies [25,26,27,29,30,32,33,35].

To complement the tabular summary, Figure 2 provides a visual synthesis of study characteristics and outcomes. The top-left figure shows that reported diagnostic performance was consistently high across included studies, with most achieving accuracy or AUC above 90% (the best value was reported for the figure, and no ablations were included). The top-right figure highlights the predominance of support vector machines and deep learning, alongside smaller contributions from ensemble and Bayesian methods. The bottom-left figure illustrates the temporal increase in LMIC-focused AI publications, with a clear rise after 2018. The bottom-right figure demonstrates the geographic concentration of studies in a few regions, particularly Iran and Sub-Saharan Africa, underscoring the need for broader representation across LMICs. Collectively, these visualizations contextualize both the promise and the current limitations of AI in cardiovascular imaging for resource-limited settings.

### 3.2. Study Selection Process

**Figure 3:**
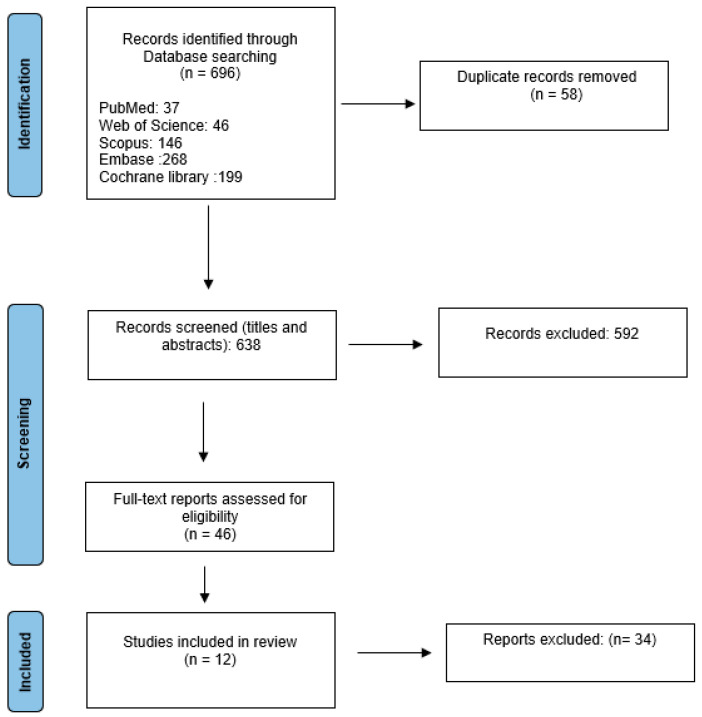
PRISMA flow chart.

Of 696 studies retrieved (37 from PubMed, 46 from Web of Science, 146 from Scopus, 199 from Cochrane Library, and 268 from Embase), 58 duplicates were removed, leaving 638 for title and abstract screening. After screening, 46 underwent full-text review, and 12 met the inclusion criteria for the final systematic review (Figure 3). The 12 included studies comprised 10 original studies on AI-based diagnostics, 1 scoping review with case examples, and 1 feasibility pilot. In addition to studies evaluating AI in cardiac imaging in low-resource environments, our review included manuscripts using datasets collected in LMICs, authored by LMIC researchers, and one conducted in remote rural Australia, a high-income setting that mirrors LMIC challenges and thus meets our low-resource criteria. We also included a review presenting clinical case examples from the Emergency Department at Port Shepstone Regional Hospital, PSRH ED, in rural KwaZulu Natal, South Africa.

### 3.3. Types of Low-Resource Settings

We defined two groups: studies in low- and middle-income countries (LMICs) and those in rural or remote areas of high-income countries with similar resource constraints. LMICs were classified per World Bank 2025 fiscal thresholds: low income (GNI per capita ≤ USD 1145), lower middle income (USD 1146–4515), and upper middle income (USD 4516–14,005) [36]. Ten studies (83%) were conducted in LMICs and two (17%) in rural or remote settings. Despite facing comparable infrastructure and workforce challenges, remote high-income areas were included under a broad low-resource category. The largest share, 33%, originated from Iran; other locations included Vietnam, Lesotho, Uganda, Tunisia, Pakistan, rural Australia, South Africa, and Ethiopia.

### 3.4. Application of AI in Cardiovascular Imaging

The research highlighted the revolutionary potential of AI and ML in cardiovascular imaging, especially in LMICs. AI-driven techniques have exhibited remarkable diagnostic precision, enhanced accessibility, and less dependence on invasive procedures like coronary angiography.

#### 3.4.1. Diagnostic Performance Across Studies

Coronary Artery Disease (CAD)

Multiple studies utilized the Z-Alizadeh Sani dataset to develop non-invasive AI-based models for detecting CAD.

- Alizadehsani et al. (2013, 2018) [25,26,27] achieved up to 96.4% accuracy and 100% sensitivity on echocardiography using SVMs with advanced feature engineering, whereas their earlier methods had a lower accuracy (79.5% LAD, 61.5% LCX, 69.0% RCA), underscoring the impact of feature selection and algorithm choice. Overall, Sequential Minimal Optimization, SVMs, and ensemble models consistently outperformed conventional techniques.
- Sayadi et al. (2022) [30] similarly achieved 95.4% accuracy and 95.9% sensitivity for early CAD detection with a diminished feature set on echocardiography, highlighting the feasibility of streamlined models in resource-limited settings.

Myocardial Infarction (MI) and Left Ventricular Hypertrophy (LVH)

Several investigations explored AI-driven echocardiography for different conditions, demonstrating robust diagnostic performance despite the limited availability of trained sonographers in LMICs.

- Nguyen et al. (2023) [32] used ensemble learning for myocardial infarction (MI) detection and reported an F1 score of 0.942 on a public dataset, although external validation on local patients saw a performance drop to 76.7% accuracy.
- Firima et al. (2024) [33] and Soh et al. (2024) [34] evaluated AI-assisted echocardiography for LVH and valve disease diagnoses. Firima et al. found 87.5% of images met the quality criteria for reliable interpretation, while Soh et al. reported diagnostic quality in over 70% of scans by non-expert operators, demonstrating that AI guidance can maintain accuracy in low-resource settings.

Rheumatic Heart Disease (RHD)

AI-based methods for rheumatic heart disease detection showed promising sensitivity and specificity without requiring invasive procedures.

- Asmare et al. (2021) [29] developed a phonocardiogram-based screening model with 96% sensitivity and specificity, providing an inexpensive, non-invasive tool suited for large-scale RHD screening programs in endemic regions.
- Brown et al. (2024) [20] reported an AUC of 0.93 using SVM and 0.84 for a DL ensemble, focusing on automated mitral regurgitation detection form Doppler echocardiography in RHD. This approach further reduced reliance on specialist echocardiographers.

Valvular Emergencies and Other Conditions

- Ekambaram et al. (2023) [31] introduced a Bayesian-inspired diagnostic framework for acute left-sided valve emergencies, leveraging handheld point-of-care echocardiography (HoPE). Despite limited Doppler capabilities, the method significantly improved the time to diagnosis in resource-constrained emergency settings.

Overall, ML algorithms, particularly SVM, ensemble methods, and DL techniques, showed high diagnostic performance, with accuracies from mid-70% to over 95% and sensitivities often above 90%. These results highlight AI’s potential to improve diagnostic precision in low-resource settings with limited invasive options and specialist expertise.

#### 3.4.2. Clinical Implications in LMICs

Task Shifting

AI tools reduce reliance on specialists. Nurse assistants using AI-assisted handheld ultrasound captured diagnostic-quality images in 87.5% of cases [33], and nurse-led AI-guided POCUS achieved 92% sensitivity for cardiac dysfunction in the CUMIN pilot [35], expanding access, freeing specialists, and addressing technologist shortages.

Early Detection and Screening

AI-guided transthoracic echocardiography found unrecognized cardiac anomalies in over 42% of remote Australian participants [34], a setting analogous to LMICs. In emergency settings, portable AI-driven echo streamlined acute valvular diagnoses [31].

Maternal and Neonatal Health

AI-based modeling of Doppler ultrasound data predicted adverse perinatal outcomes in Pakistan, enabling earlier intervention in high-risk pregnancies [28].

Improving Accessibility and Scalability

AI-enhanced phonocardiogram-based RHD screening and portable echocardiography achieved high diagnostic accuracy at low cost [20,29], supporting scalable community screening programs where advanced imaging is scarce.

Potential for Cost Effectiveness

Although few studies assessed economics, use of portable devices, minimal operator training, and reduced invasive procedures suggest long-term cost savings. Overall, AI-driven imaging can lower barriers to specialized diagnostics, streamline early detection, boost efficiency, and enable less-trained staff to deliver high-quality care. Despite challenges in operator training, data infrastructure, and regulation, evidence supports AI-based imaging as a viable, potentially transformative approach to reducing cardiovascular disease in LMICs.

#### 3.4.3. Challenges and Barriers

Several recurring obstacles to AI implementation in cardiovascular imaging were identified across the 12 included studies (Table 2, Table 3 and Table 4).

1. Limited Dataset Diversity and Small Sample Sizes
  ○ Nguyen et al. [32] reported reduced accuracy when moving from a public dataset to local patient data, indicating that algorithms trained on narrowly representative datasets may underperform in external validation.
  ○ Sayadi et al. and Firima et al. [30,33] also highlighted the need for larger, multi-center datasets to improve generalizability.
2. Operator Dependence
  ○ Ekambaram et al. and Tromp et al. [31,35] noted that the quality of image acquisition was influenced by the skill level of the individual performing the scan, potentially affecting diagnostic accuracy.
  ○ Soh et al. [34] similarly reported that non-expert operators sometimes struggled to obtain complete echocardiographic views, resulting in inconclusive scans in a minority of cases.
3. Infrastructure Constraints
  ○ Firima et al. and Asmare et al. [29,33] both mentioned access to portable ultrasound devices and the reliability of power sources as significant barriers, particularly in rural or remote environments.
  ○ Hoodbhoy et al. [28] noted that data collection and storage require stable networks, which can be challenging in LMIC settings.
4. Algorithm Generalizability
  ○ Brown et al. and Alizadehsani et al. [20,26] indicated concerns regarding whether models trained on single-center data would be robust enough when applied to different populations or imaging devices (concern over generalizability).
  ○ Nguyen et al. [32] specifically mentioned high computational demands that could limit algorithm deployment on lower-end hardware available in LMIC settings.
5. Reliance on Protocol or Synthetic Data
  ○ Hoodbhoy et al. and Sayadi et al. [28,30] used partly protocol-driven or synthetic datasets for model training, which may not reflect real-world heterogeneity. This constraint can impede immediate clinical adoption of the AI models.
6. Miscellaneous Factors
  ○ While some studies, such as Asmare et al. [29], addressed financial and policy considerations in passing, direct cost analyses or detailed funding barriers were not comprehensively reported.
  ○ No included study performed a formal ethical or regulatory evaluation, though several (Brown et al., Tromp et al.) [20,35] the broader implications of data governance and patient privacy in resource-limited contexts.

Overall, the included studies consistently cited data limitations, operator training, and infrastructure as the most pressing challenges to implementing AI-based cardiovascular imaging in low-resource settings. The extent to which these barriers affected study outcomes varied, but most authors emphasized the need for larger, context-specific datasets and robust training programs to fully realize AI’s potential in LMICs.

#### 3.4.4. Future Directions

Several studies offered recommendations to advance the use of AI in cardiovascular imaging within low-resource settings:

1. Expanding and Diversifying Datasets
  ○ Nguyen et al. [32] and Brown et al. [20] emphasized the need for larger, more-representative datasets to enhance generalizability across different LMIC populations. They suggested multi-center data sharing as a strategy to mitigate overfitting and improve algorithm robustness.
2. Multi-Center Validation and Prospective Trials
  ○ Sayadi et al. [30] and Tromp et al. [35] highlighted the importance of conducting prospective, multi-center validation studies to confirm the real-world effectiveness of AI tools beyond single-site or protocol-driven data.
3. Improving Algorithm Adaptability
  ○ Firima et al. [33] noted that ML models should be optimized to handle variable imaging quality and operator experience. They proposed iterative algorithm training using feedback loops from actual clinical deployment to ensure sustained performance in resource-limited environments.
4. Capacity Building and Workforce Training
  ○ Soh et al. [34] and Ekambaram et al. [31] recommended investing in targeted training programs for healthcare workers at different skill levels, from nurses and technologists to general practitioners, enabling more-consistent image acquisition and interpretation in remote settings.
5. Infrastructure and Policy Support
  ○ Asmare et al. [29] and Hoodbhoy et al. [28] acknowledged the importance of infrastructure development, including reliable power, internet connectivity, and maintenance of portable devices. They also noted the need for supportive policies and regulatory frameworks to facilitate funding, data governance, and ethical implementation of AI technologies.

Overall, the included studies collectively underscore the need for larger, collaborative research efforts and sustained investments in training and infrastructure. These measures were seen as critical to extending AI’s benefits in cardiovascular imaging across varied LMIC contexts and ensuring long-term scalability of these innovations [37,38,39].

## 4. Discussion

This review reveals AI’s promise for improving cardiovascular imaging in LMICs by demonstrating the high diagnostic efficacy of AI-driven modalities alongside the barriers to sustainable deployment in resource-limited settings. The included studies report accuracies often above 90% for CAD, MI, and RHD. Many leveraged machine learning and deep learning to enable task shifting, early detection, and greater access in rural or remote regions. Despite these advances, common challenges remain in operator training, infrastructure constraints, and lack of diverse datasets.

**Diagnostic Performance and Clinical Impact**

AI showed strong results for CAD, MI, and RHD in LMIC settings. Adding image features to traditional risk factors improved CVD and stroke risk classification [40]. Alizadehsani et al. (2013, 2018) [25,26] used SMO and SVM to reach 96.4% accuracy, potentially reducing invasive angiography [41]. AI-driven echo aids scarce specialists: Firima et al. [33] found non-expert operators using portable ultrasound with deep learning achieved 87.5% evaluable images, and Asmare et al. [29] reported a phonocardiogram-based RHD screen with 96% sensitivity and specificity for large-scale LMIC use [42].

**Decentralization and Task Shifting**

AI-guided echo by non-experts (Tromp et al., Soh et al.) [34,35] shifts specific diagnostics from specialists to frontline workers, easing cardiologist workload and boosting rural access, in line with universal health coverage goals [42].

**Time and Workforce Savings**

FDA-approved CMR software automates ventricular segmentation in under 10 s, illustrating how cloud-based AI streamlines workflows [4]. At the 2023 RAD AID Conference, AI was highlighted for worklist triage, risk stratification, structured reporting, and quality assurance to address radiology shortages in LMICs [43].

**Barriers to Implementation**

Key challenges include limited dataset diversity, operator dependence, infrastructure gaps, funding limits, and ethical legal concerns. HIC-trained models underperform in LMIC populations [9,44], as Nguyen et al. found lower accuracy on Vietnamese data [45,46,47]. Operator variability affects image quality [48,49,50]. Unreliable power, poor internet, and scarce devices impede deployment [51,52], and maintaining cloud-based storage can be difficult [53,54]. Funding often favors infectious diseases [55], while unclear ethical and regulatory guidelines raise privacy and transparency issues [19,43,56]. Overcoming these barriers requires multi-center data sharing, standardized training, targeted infrastructure investment, and robust policy development.

**Lessons from AI Applications in Cardiac MRI and CT (HIC Experience)**

Echocardiography was the primary modality in LMIC AI studies, while advanced cardiac MRI (CMR) and cardiac CT (CCT) are more common in high-income settings. Our review identified no LMIC-focused CMR or CCT studies. The following section therefore draws on the HIC literature to provide a concise overview of key applications, benefits, and challenges. These insights are presented as perspectives on potential but speculative pathways for LMIC adoption whose feasibility depends on local validation, infrastructure, and governance.

**Cardiac Magnetic Resonance (CMR)**

- **Accelerated Acquisition and Reconstruction:** DL, particularly CNNs, has sped up scans and reconstructed under-sampled data, even enabling real-time CMR via compressed sensing plus DL, without sacrificing accuracy [57,58]. Shorter exam times could improve scanner access in LMICs.
- **Automated Segmentation:** Architectures such as the U-Net automate LV and RV blood-pool/endocardium and myocardium delineation with high precision, saving time and reducing inter-observer variability in settings with few specialists [59,60].
- **Myocardial Tissue Characterization:** AI algorithms detect and quantify fibrosis, edema, or infarction on LGE and T1/T2 maps, and ML texture analysis distinguishes myocardial pathologies objectively [61,62], supporting early intervention.
- **Risk Stratification and Prognostication:** AI models integrate CMR data with clinical parameters for personalized CAD and cardiomyopathy risk predictions [63]. Emerging frameworks can automate scan parameters to lower operator dependency [64]. Effective LMIC deployment will require diverse data, local validation, and robust infrastructure and policy support.

**Cardiac Computed Tomography (CCT)**

- **Acquisition, Reconstruction, and Radiation Reduction:** AI optimizes low-dose CCT protocols, enhancing image quality while cutting radiation exposure [65]. LMICs could partially adopt these techniques with infrastructure or cloud-based solutions.
- **CAD Detection and Plaque Characterization:** AI-augmented CCTA improves sensitivity and specificity for stenosis and enables quantitative plaque assessment [66]. The CLARIFY study showed strong overall performance but noted variability in high-risk plaque feature detection [66,67,68]. Such tools could expand non-invasive CAD diagnostics in LMICs if software and data capabilities permit.
- **Structural Heart Segmentation:** Automated segmentation of chambers and vessels guides procedures like TAVI, accelerating planning and reducing variability [69]. LMIC tertiary centers could streamline interventions with these tools.
- **Challenges and Future Directions:** Limited LMIC data, unclear regulations, and “black box” concerns hinder CCT AI adoption. Future efforts should focus on local validation, data governance, and development of models that predict plaque instability to prevent acute events.

**Figure 4:**
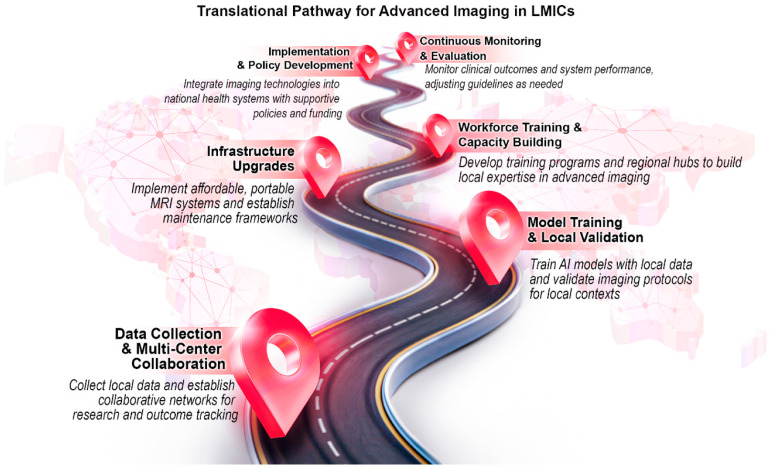
Translational pathway for AI implementation in LMICs.

**Overarching Lessons for LMIC Adoption (Figure 4)**

Drawing on HIC experiences with advanced imaging (CMR and CCT), several guiding principles emerge for LMICs:

1. Gradual Implementation
  ○ Real-time CMR or advanced plaque quantification may be too resource intensive for a broad LMIC rollout. Smaller trials or simple AI segmentation can still yield benefits.
2. Local Data and Validation
  ○ HIC algorithms require retraining or validation on LMIC cohorts, using global pre-trained models with local fine tuning.
  ○ RAD AID recommends phased AI introduction, i.e., a local tool validation against existing practice, stakeholder partnerships, super-user training, and workflow integration, to ensure sustainability [8,13,70].
3. Infrastructure and Cost Effectiveness
  ○ Strategies that reduce scan time and operator dependence address LMIC needs but start-up hardware and software costs must be balanced against long-term labor savings and improved diagnostics.
  ○ With limited radiology expertise in LMICs, investment in workforce education and development is essential [6,70].
4. Ethical and Regulatory Frameworks
  ○ Transparent, explainable AI is essential for trust in low-technology medical settings; clear rules on data ownership, privacy, and liability are needed to safeguard patients.
  ○ Governments must add AI education to medical training and promote collaboration between HICs and LMICs to build capacity in resource-limited settings.
  ○ Resource-limited health institutions have low participation in AI development; increasing their involvement is critical to produce tools validated for diverse global populations [8].
5. Collaborations and Funding
  ○ Partnerships between local institutions, industry, academia, and NGOs can channel resources toward pilot programs that demonstrate cost effectiveness and scalability, eventually guiding policy decisions to invest in advanced imaging solutions for the wider populace [69].

AI in cardiac MRI and CT in high-income countries has improved acquisition speed, diagnostic accuracy, and workflows, helping to relieve staff shortages and reduce bottlenecks. By training local operators, using AI algorithms developed on data from both HI and LMI countries and adapting workflows to match infrastructure constraints, LMICs can modernize older, slower methods and enhance cardiovascular care. However, safe and effective deployment requires robust multi-center data collection, local validation, upgraded infrastructure, and strong policy support in resource-limited settings with a high disease burden.

**Figure 5:**
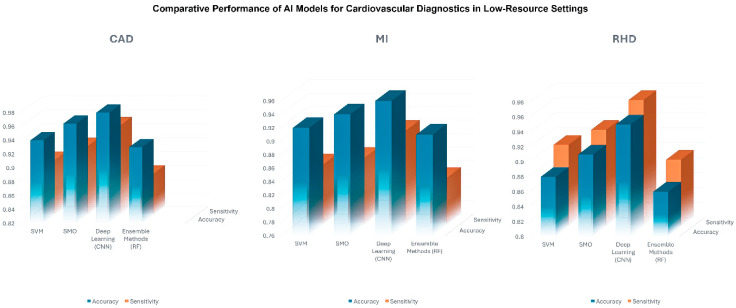
Comparative performance of AI models.

**Comparing AI Models (Figure 5)**

Choosing an optimal ML approach remains a key challenge for both LMIC and HIC applications in cardiovascular imaging, and there are multiple algorithms to choose from [71]. While DL architectures such as CNNs, specifically U-Net, often excel in advanced modalities like CMR or CCT [1，^2^，^3^，^4^，^5^] simpler ML techniques have also demonstrated robust performance particularly in echocardiography for LMIC settings.

A comprehensive evaluation of ML-based CAD diagnosis (1992–2019) found that KNN, ANN, and SVM were commonly the highest-performing classifiers [1]. Studies included in our systematic review likewise showed that Sequential Minimal Optimization (SMO) and SVM could achieve 94–96% accuracy for CAD detection when combined with feature engineering, e.g., Alizadehsani et al. [25] Ensemble methods (e.g., bagging, boosting) further enhanced diagnostic precision, especially in cases with complex, high-dimensional data.

However, model selection is highly context dependent:

- Data Volume and Quality
  In LMICs often constrained by small or non-diverse datasets, simpler algorithms like Naïve Bayes, decision trees, or linear SVM may be more practical but risk underfitting. More-complex architectures (e.g., CNNs, transformers) can excel with large, high-quality datasets prevalent in HIC research but may falter in low-resource environments without sufficient data or computing power.
- Computational Infrastructure
  Deploying advanced DL models for CMR or CCT typically demands GPU acceleration, stable electricity, and robust IT support, and such resources may be limited in LMIC contexts. Hence, moderate-complexity algorithms (e.g., ensemble trees) might strike a balance between accuracy and feasibility.
- Clinical Application
  Automated segmentation in MRI or CT often relies on U-Net-based frameworks, while echocardiography in LMICs frequently uses SVM or random forests for classification tasks (RHD detection, LVH identification). The task at hand, be it segmenting structures, identifying stenosis, or stratifying risk, should guide which model best balances interpretability, speed, and accuracy.

Ultimately, no single model universally outperforms across all cardiovascular imaging tasks. Each must be tailored to the local setting, available data, and clinical requirements, underscoring the importance of algorithmic flexibility and iterative validation in LMICs and HICs alike.

**Limitations Encountered**

This review should be interpreted considering several limitations. The included studies were heterogeneous in design, dataset size, and outcome measures, precluding meta-analysis. Most studies were single-center and lacked external validation, raising concerns about generalizability. Furthermore, publication bias cannot be excluded, as negative or inconclusive studies are less likely to be reported. At the review level, only English-language publications were included, which may have limited the capture of relevant work from non-English-speaking LMICs.

**Future Directions and Recommendations**

Addressing the challenges and harnessing the lessons from both LMIC echocardiography implementations and HIC MRI/CT research will require targeted, collaborative efforts. While our recommendations focus on data, workforce, infrastructure, governance, and collaborations, reviewers have rightly noted that they must move beyond general principles to include specific and applicable strategies. To that end, we expand each point below with actionable steps:

1. Strengthening Data Repositories
  - Pool anonymized imaging across LMIC centers through regional hubs.
  - Establish standardized imaging protocols and metadata collection to facilitate cross-site harmonization.
  - Fine-tune HIC-trained models on smaller local samples using federated learning or transfer learning to reduce reliance on large, centralized datasets.
2. Building Workforce Capacity
  - Develop modular AI curricula integrated into medical and allied health training programs, with tiered certifications for nurses, technicians, and general practitioners.
  - Train a cadre of “super-users”—clinicians or technicians at regional hospitals who receive advanced training in AI imaging tools. These individuals serve as local experts who provide mentorship, ensure quality control, and support surrounding facilities with troubleshooting and guidance.
  - Deploy AI systems with built-in quality control dashboards that provide immediate feedback to novice operators, further reducing variability.
3. Scalable Infrastructure and Technology
  - Prioritize portable and battery-operated imaging devices with integrated AI, particularly for rural outreach.
  - Explore hybrid cloud–edge computing solutions to overcome intermittent internet access, where data are pre-processed locally and synced centrally when connectivity allows.
  - Develop cost-effectiveness frameworks that balance upfront hardware costs against long-term reductions in invasive testing and workforce strain.
4. Ethical, Legal, and Regulatory Frameworks
  - Establish regional regulatory sandboxes in LMICs to allow pilot testing of AI tools under controlled conditions while governance frameworks are developed.
  - Incorporate explainability modules (e.g., heatmaps, decision trees) into AI outputs to build clinician trust.
  - Create open-source template policies for data governance, privacy, and algorithmic accountability that can be adapted by LMIC ministries of health.
5. Cross-Sector Collaborations
  - Incentivize joint programs between local universities, NGOs, and private companies to run small-scale pilots (e.g., AI-assisted RHD screening in schools, nurse-led CMR segmentation in tertiary centers).
  - Secure donor and government funding specifically tied to measurable outcomes such as reduction in time to diagnosis or an increase in number of patients screened rather than generic “AI capacity building.”
  - Launch multi-center prospective trials evaluating diagnostic accuracy, cost effectiveness, and patient outcomes across diverse LMIC populations to guide scale-up.

By adopting these strategies, stakeholders can scale from echo-based AI toward advanced modalities like CMR and CCT in a stepwise, context-sensitive manner. This approach ensures that innovations developed in HICs are translated into LMICs not as speculative possibilities but as rigorously validated, sustainable, and equitable healthcare solutions.

## 5. Conclusions

AI-based cardiovascular imaging offers strong potential to improve diagnostic accuracy, efficiency, and access across imaging modalities. In LMICs, echocardiography is the most-studied platform, enabling task shifting and early detection. Advances from HICs in cardiac MRI and CT, such as automated segmentation and risk assessment, suggest future opportunities for under-resourced settings.

Realizing this potential of AI requires robust datasets, tailored models, infrastructure, and ethical oversight. By addressing these needs and learning from both LMIC and HIC experiences, health systems can close care gaps and advance global cardiovascular equity.

## Figures and Tables

**Table 1:** Inclusion and exclusion criteria.

Inclusion Criteria	Exclusion Criteria
Conducted in LMICs as defined by the 2022–2023 World Bank Classification or in rural/remote settings in high-income countries Original research articles Used at least one imaging modality for cardiac imaging (e.g., echocardiography, cardiac MRI) Employed AI techniques in the study (see AI definition below) Published in English	Reviews, editorials, conference abstracts, or case reports Focused solely on non-cardiac imaging Conducted in HICs (with the exception of rural or remote low-resource settings within high-income countries) Not AI related Not English

## Data Availability

All data analyzed in this study were obtained from publicly available sources. The extracted data supporting the findings of this systematic review are available from the corresponding author upon reasonable request.

## Supplementary Materials

The following supporting information can be downloaded at https://www.mdpi.com/article/10.3390/bioengineering12101038/s1, Table S1: title. The Supplementary Materials provide the complete literature search strategy used for this review.
