# Supplementary material for: From Echocardiography to CT/MRI: Lessons for AI Implementation in Cardiovascular Imaging in LMICs—A Systematic Review and Narrative Synthesis
Source: Bioengineering (Basel). 2025 Sep 27;12(10):1038. doi: 10.3390/bioengineering12101038 (PMC12561239; doi:10.3390/bioengineering12101038)
Supplement: Supplementary file 1 [file bioengineering-12-01038-s001.zip › bioengineering-3875870-supplementary.pdf]

Database	Search strategy	Results	
1)PubMed<br><br>(To January 5, 2025)	#1	(("cardiovascular"[Title/Abstract] OR "cardiac"[Title/Abstract] OR "heart"[Title/Abstract] OR "Cardiovascular Diseases/diagnostic imaging"[Mesh])	
	#2	("imaging"[Title/Abstract] OR "echocardiography"[Title/Abstract] OR "Magnetic Resonance Imaging"[Mesh] OR "Tomography, X-Ray Computed"[Mesh] OR "CT scan"[Title/Abstract] OR "computed tomography"[Title/Abstract] OR "angio*"[Title/Abstract] OR "MRI"[Title/Abstract]))	
	#3	(("artificial intelligence"[Title/Abstract] OR "ai"[Title/Abstract] OR "radiomic*"[Title/Abstract] OR "machine learning"[Title/Abstract]))	
	#4	(("low income countr*"[Title/Abstract] OR "middle income countr*"[Title/Abstract] OR "Developing Countries"[Mesh]<br>OR (Afghanistan[Title/Abstract]) OR (Burkina Faso[Title/Abstract]) OR (Burundi[Title/Abstract]) OR (Central African Republic[Title/Abstract])<br>OR (Chad[Title/Abstract]) OR (Congo[Title/Abstract]) OR (Eritrea[Title/Abstract]) OR (Ethiopia[Title/Abstract]) OR (Gambia[Title/Abstract])<br>OR (Korea[Title/Abstract]) OR (Liberia[Title/Abstract]) OR (Madagascar[Title/Abstract]) OR (Malawi[Title/Abstract]) OR (Mali[Title/Abstract])<br>OR (Mozambique[Title/Abstract]) OR (Niger[Title/Abstract]) OR (Rwanda[Title/Abstract]) OR (Sierra Leone[Title/Abstract]) OR (Somalia[Title/Abstract])<br>OR (South Sudan[Title/Abstract]) OR (Sudan[Title/Abstract]) OR (Uganda[Title/Abstract]) OR (Togo[Title/Abstract]) OR (Yemen[Title/Abstract])<br>OR (Syria[Title/Abstract]) OR (India[Title/Abstract]) OR (Iran[Title/Abstract]) OR (Algeria[Title/Abstract]) OR (Egypt[Title/Abstract])<br>OR (Jordan[Title/Abstract]) OR (Lebanon[Title/Abstract]) OR (Mongolia[Title/Abstract]) OR (Morocco[Title/Abstract]) OR (Nigeria[Title/Abstract])<br>OR (Pakistan[Title/Abstract]) OR (Philippines[Title/Abstract]) OR (Senegal[Title/Abstract]) OR (Tajikistan[Title/Abstract])<br>OR (Tunisia[Title/Abstract]) OR (Ukraine[Title/Abstract]) OR (Uzbekistan[Title/Abstract]) OR (Vietnam[Title/Abstract])<br>OR (Zimbabwe[Title/Abstract])))	
	#5	#1 AND #2 AND #3 AND #4	37
2) Embase<br><br>(To January 5, 2025)	#1	(('cardiovascular':ab,ti OR 'cardiac':ab,ti OR 'heart':ab,ti OR 'cardiovascular diseases':ab,ti)	
	#2	'imaging':ab,ti OR 'echocardiography':ab,ti OR 'magnetic resonance imaging':ab,ti OR 'ct scan':ab,ti OR 'computed tomography':ab,ti OR 'x-ray tomography':ab,ti OR 'mri':ab,ti OR 'angiography':ab,ti OR 'angio':ab,ti OR 'ct':ab,ti)	
	#3	('artificial intelligence':ab,ti OR 'ai':ab,ti OR 'radiomics':ab,ti OR 'machine learning':ab,ti)	
	#4	('low income countries':ab,ti OR 'middle income countries':ab,ti OR 'developing countries':ab,ti OR 'afghanistan':ab,ti OR 'burkina faso':ab,ti OR 'burundi':ab,ti OR 'central african republic':ab,ti OR 'chad':ab,ti OR 'congo':ab,ti OR 'eritrea':ab,ti OR 'ethiopia':ab,ti OR 'gambia':ab,ti OR 'korea':ab,ti OR 'liberia':ab,ti OR 'madagascar':ab,ti OR 'malawi':ab,ti OR 'mali':ab,ti OR 'mozambique':ab,ti OR 'niger':ab,ti OR 'rwanda':ab,ti OR 'sierra leone':ab,ti OR 'somalia':ab,ti OR 'south sudan':ab,ti OR 'sudan':ab,ti OR 'uganda':ab,ti OR 'togo':ab,ti OR 'yemen':ab,ti OR 'syria':ab,ti OR 'india':ab,ti OR 'iran':ab,ti OR 'algeria':ab,ti OR 'egypt':ab,ti OR 'jordan':ab,ti OR 'lebanon':ab,ti OR 'mongolia':ab,ti OR 'morocco':ab,ti OR 'nigeria':ab,ti OR 'pakistan':ab,ti OR 'philippines':ab,ti OR 'senegal':ab,ti OR 'tajikistan':ab,ti OR 'tunisia':ab,ti OR 'ukraine':ab,ti OR 'uzbekistan':ab,ti OR 'vietnam':ab,ti OR 'zimbabwe':ab,ti)	
	#5	#1 AND #2 AND #3 AND #4	268
3)Cochrane Library<br><br>(To January 5, 2025)	#1	("cardiovascular" OR "heart")	
	#2	("imaging" OR "MRI" OR "echocardiography" OR "CT scan")	
	#3	("artificial intelligence" OR "machine learning")	
	#4	#1 AND #2 AND #3	199

Database	Search strategy	Results	
4) Scopus<br><br>(To January 5, 2025)	#1	TITLE-ABS-KEY(("cardiovascular" OR "cardiac" OR "heart")	
	#2	("imaging" OR "echocardiography" OR "Magnetic Resonance Imaging" OR "Tomography, X-Ray Computed" OR "CT scan" OR "computed tomography" OR "angio*" OR "MRI")	
	#3	("artificial intelligence" OR "AI" OR "radiomic*" OR "machine learning")	
	#4	("low income countr*" OR "middle income countr*" OR "Developing Countries"<br>OR "Afghanistan" OR "Burkina Faso" OR "Burundi" OR "Central African Republic" OR "Chad" OR "Congo" OR "Eritrea" OR "Ethiopia"<br>OR "Gambia" OR "Korea" OR "Liberia" OR "Madagascar" OR "Malawi" OR "Mali" OR "Mozambique" OR "Niger" OR "Rwanda"<br>OR "Sierra Leone" OR "Somalia" OR "South Sudan" OR "Sudan" OR "Uganda" OR "Togo" OR "Yemen" OR "Syria" OR "India"<br>OR "Iran" OR "Algeria" OR "Egypt" OR "Jordan" OR "Lebanon" OR "Mongolia" OR "Morocco" OR "Nigeria" OR "Pakistan"<br>OR "Philippines" OR "Senegal" OR "Tajikistan" OR "Tunisia" OR "Ukraine" OR "Uzbekistan" OR "Vietnam" OR "Zimbabwe"))	
	#5	#1 AND #2 AND #3 AND #4	146
5) Web Of Science<br><br>(To January 5, 2025)	#1	TS=(("cardiovascular" OR "cardiac" OR "heart")	
	#2	("imaging" OR "echocardiography" OR "Magnetic Resonance Imaging" OR "Tomography, X-Ray Computed" OR "CT scan" OR "computed tomography" OR "angio*" OR "MRI")	
	#3	("artificial intelligence" OR "AI" OR "radiomic*" OR "machine learning")	
	#4	("low income countr*" OR "middle income countr*" OR "Developing Countries"<br>OR "Afghanistan" OR "Burkina Faso" OR "Burundi" OR "Central African Republic" OR "Chad" OR "Congo" OR "Eritrea" OR "Ethiopia"<br>OR "Gambia" OR "Korea" OR "Liberia" OR "Madagascar" OR "Malawi" OR "Mali" OR "Mozambique" OR "Niger" OR "Rwanda"<br>OR "Sierra Leone" OR "Somalia" OR "South Sudan" OR "Sudan" OR "Uganda" OR "Togo" OR "Yemen" OR "Syria" OR "India"<br>OR "Iran" OR "Algeria" OR "Egypt" OR "Jordan" OR "Lebanon" OR "Mongolia" OR "Morocco" OR "Nigeria" OR "Pakistan"<br>OR "Philippines" OR "Senegal" OR "Tajikistan" OR "Tunisia" OR "Ukraine" OR "Uzbekistan" OR "Vietnam" OR "Zimbabwe"))	
	#5	#1 AND #2 AND #3 AND #4	46
Total			696

Supplementary Table S1. Search Strategy.
